# The Peer Effect on Dietary and Nutritional Cognition among Primary School Students

**DOI:** 10.3390/ijerph19137727

**Published:** 2022-06-23

**Authors:** Lei Gao, Ruotong Li, Peiyan Zhao, Ying Zhang

**Affiliations:** 1 School of Economics and Management, Shi Hezi University, Shi Hezi 832061, China; 18099117785@163.com; 2 College of Economics and Management, China Agricultural University, Beijing 100083, China; liruotong_cau@163.com (R.L.); zhaopeiyan333@163.com (P.Z.)

**Keywords:** peer effect, dietary cognition, nutritional cognition, primary school students

## Abstract

This study uses data from a 2018 survey of 11,384 students in five Chinese provinces to investigate the peer effect on students’ dietary and nutritional cognition. Children’s eating habits have an important impact on their growth and health. Studies have shown that students’ dietary behavior is mainly affected by their dietary and nutritional cognition. Therefore, studying the influencing factors of elementary school students’ cognition of diet and nutrition has become an important research question. However, there are few discussions about the impact of peers’ dietary and nutritional cognition on students’ cognition of diet and nutrition. Consequently, this paper studied the peer effect on students’ cognition of diet and nutrition. The results indicated that peers had a significant impact on the students’ dietary and nutritional cognition. The endogeneity problem was solved using peers’ parents’ dietary and nutritional cognition scores and average educational level as instrumental variables. The impact of peer cognition on diet and nutrition was heterogeneous among different groups. The significance and degree of the peer effect differed based on peer relations, gender, age and school. The results indicated that in addition to family, school, teachers and other factors, peers were an important influencing factor.

## 1. Introduction

Diet and nutrition in childhood are of great significance to the construction of early healthy human capital. In recent years, different countries have implemented improvement plans and measures for children’s nutritional and health status and achieved good results; however, there are still concerns about children’s eating behavior in some countries. The 2019 Global School-Based Student Health Survey (GSHS) collected nearly thirty-day fruit and vegetable intake among students aged 13–15 in seventy-three countries, and the results showed that about half of the countries reported that 10–30% of students did not eat any fruit, and a quarter reported that 10–30% of students did not eat any vegetables in thirty days. [1]. Nearly 70% of countries reported that at least half of students eat fast food every week, and all countries found that at least one in five students drink carbonated soft drinks once a day [1]. Research shows that the average intake of fruits and vegetables for children (aged 4–12) in Ireland, the United Kingdom and the Netherlands is 221–272g per day, which is lower than the WHO recommendation of at least 400g per day [2,3,4]. In 2018, a survey of 507 primary school fifth-graders in Beijing, China found that 80.3% and 67.8% of children ate vegetables or fruits 6–7 days a week, while the intake of meat, eggs, milk and beans was lower (57.5%, 45.3%, 60.0% and 17.3% respectively), and 12.7–22.1% of students ate sugary drinks and candy more than four days a week [5].

Dietary behavior is a key factor affecting children’s nutrition intake [6,7]. Childhood nutrition is essential for health and intellectual development over the life course and significantly influences the country’s economic and social growth [8,9,10]. Keeley et al. (2019) [11] found that the triple burden of malnutrition, which includes malnutrition, micronutrient deficiencies, and overweightness, is caused by poor dietary quality. In addition, compared to adults, children have higher nutrient requirements. Malnutrition in children leads to stunting, physical weight loss, and serious diseases, such as anemia [12,13]. From an economic perspective, the nutritional deficiency leads to a decline in immunity and affects intellectual development and labor ability, and it caused an economic loss estimated to account for approximately 2–3% of global GDP [14]. In developing countries, solving the problem of child malnutrition would reduce approximately one-third of the country’s disease burden [14]. Moreover, childhood overnutrition can lead to overweightness and obesity, dramatically increasing the risk of chronic diseases, such as diabetes, in adulthood [15]. The Report on the Status of Nutrition and Chronic Diseases of Chinese Residents (2020) indicated that 11.1% and 7.9% of students aged six to 17 and 6.8% and 3.6% of children under the age of six were overweight and obese, respectively.

Studies have shown that students’ knowledge of diet and nutrition can affect their dietary behavior [16,17]. A systematic review showed that food literacy may play a role in shaping adolescents’ dietary intake, with higher levels of dietary knowledge indicating a higher likelihood of maintaining healthy eating behaviors [18]. In addition, the dietary skills and behaviors learned in adolescence will continue in later life [19]. Joulaei et al. (2018) [20] found that an increase in functional nutrition literacy was associated with lower sugar intake and better energy balance in boys and higher dairy intake in girls.

Therefore, improving children’s cognition of diet and nutrition is important, and a growing number of studies have been conducted to identify parents’ and teachers’ influence on children’s dietary and nutritional cognition. Velardo and Drummond (2019) [21] demonstrated that parents and teachers were key factors affecting children’s interaction with nutrition information, and children especially emphasize their trust in their teachers as health “experts”.

Peer interaction is generally considered one of the factors that influence children’s behavioral changes [22]. Peers’ actions can lead to a positive or negative spillover effect and influence many aspects of children’s lives [23,24]. Children are more likely to copy peers than adults [25]. In addition, children are exposed to many peers of the same age and are often similar in other ways [26]. Research also shows that the eating habits of children and adolescents are affected by peer examples [27]. However, little attention has been devoted to the peer effect on children’s dietary and nutritional cognition. 

To address this gap, this study aimed to estimate the peer effect on students’ dietary and nutritional cognition using 2018 survey data of 11,384 students in Beijing, Suzhou, Henan, Anhui and Yunnan. The contributions of this paper are as follows: firstly, the endogeneity problem is solved by using instrumental variables. Secondly, through heterogeneity analysis, this paper discusses the differences of peer effect among different student groups.

## 2. Materials and Methods

The study data were derived from the HNPS (Health and Nutrition Panel survey), which was carried out by the School of Economics and Management, China Agricultural University in 2018. This study was approved by the Ethics Committee of China Agricultural University. All procedures performed in studies involving human participants were following the 1964 Helsinki declaration and its later amendments. All necessary permits have also been obtained from the Chinese government and the local education bureau. All students and their legal guardians participating in the survey fully understand the purpose of the survey and agree to participate in the project. The sample included 11,384 students from Beijing, Jiangsu, Henan, Anhui and Yunnan. Beijing and Jiangsu have high development levels, high labor demand and high wage levels. Thus, for many migrant workers, Beijing and Jiangsu are good places to find work. Furthermore, Beijing and Jiangsu are the main destinations for employment for Henan and Anhui farmers, respectively. When farmers go out to work, some will take their children, and some will leave their children in their hometown. Therefore, there are many migrant children in Beijing and Jiangsu, and there are many left-behind children in Henan, Anhui and Yunnan.

The survey used a four-stage sampling method. First, Beijing, Jiangsu, Henan, Anhui and Yunnan were selected as the survey provinces. Second, survey counties were selected in Henan, Anhui and Yunnan. The counties were divided into three grades based on per capita industrial output reported in the County Statistical Yearbook. Two counties were randomly selected in the first grade, one in the second grade and two in the third grade. Third, survey towns were selected from each sampled county. Towns were divided into three grades based on per capita industrial output. Two towns were randomly selected for each grade. The sample school was the central primary school in each selected town. Fourth, the survey class was selected from the survey-grade (5th grade and 6th grade) of the sample school. For schools with one class in each grade, this class was included. Otherwise, the sample class was selected using random sampling for each grade. All students in the selected class took part in the survey, and all students and their guardians fully understood and agreed to participate in the survey. For Beijing, Suzhou and Jiangsu, all schools that met the survey requirements were listed and thirty schools were randomly selected.

Four categories of student information were collected using the questionnaire. First, the questionnaire investigated students’ cognitive abilities, including their dietary and nutritional cognition, determined based on the nutritional health status survey of the Nutrition Improvement Program for the Rural Compulsory Education Students. The questionnaire contained seven questions (Table A1), such as “What do you think is the best food source of vitamins and minerals?”. Students received one point for each correct answer, and we take the number of correctly answered questions as the score of dietary and nutritional cognition based on the method of Zhang et al. (2012) [28]. In order to more accurately measure the nutrition cognition of students and parents, this study uses the item response theory (IRT) model to standardize the nutrition cognition score in the robustness test [29]. To measure the impact of the peer effect on students’ dietary and nutritional cognition accurately, this study standardized students’ scores. Second, the questionnaire asked students about their social communication network, with questions such as “What is the name of your best friend in your class now?” and “Why did you choose them to be your best friend?” Based on this question, peers were identified. Third, the questionnaire inquired about student characteristics, such as gender, age, height and weight. Some of these factors were selected as control variables based on previous studies. In addition, this section included questions on students’ dietary behavior and physical attributes, such as unhealthy eating behavior score and height-for-age z-score (Table 1). The unhealthy eating behavior score was calculated based on students’ review of the types of unhealthy food, such as bread, pastries, biscuits, chocolate, fried snacks (potato chips, French fries, etc.), other snacks and drinks, consumed in the past 24 h. The unhealthy eating behavior score was the sum of the types of unhealthy food students had eaten. Fourth, the questionnaire inquired about students’ families, including parents’ dietary and nutritional cognition and education. Similar to students’ dietary and nutritional cognition scores, parents’ scores were standardized. The family situation questionnaire was completed by the guardian after the student took it home.

The summary statistics are reported in Table 1. The results revealed that the dietary and nutritional cognition of the sample students was low, with an average score of 2.52, indicating that among seven dietary and nutritional cognition questions, the average number of correct answers per student was less than three. Yaghi (2022) [30] also found that students’ dietary cognition level is very low. Parents’ dietary and nutritional cognition was also very low, with an average of 1.33 points. Studies have found that parents’ dietary nutrition cognition is related to their education level [31]. The average educational level of parents in this study is only nine years, and the educational level is very low. In addition, there were significant problems in students’ dietary behavior. Overall, each student ate nearly three kinds of unhealthy food in the past 24 h. Moreover, there were slightly more boys than girls in the sample. The average age of the sample students was 11 years. The overall average educational level of parents was approximately nine years, with the education of fathers generally higher than that of mothers. In addition, fathers were generally older than mothers.

**Table 1:** Variables, variable names and summary statistic.

Variables	Definition	Mean	Min	Max	SD	Obs
Score of dietary and nutritional cognition	student’s cognition of diet and nutrition	2.52	0	7	1.504	11,384
Gender dummy	Dummy; 1 = boy; 0 = girl	0.53	0	1	0.499	11,384
Age	Age measured by year	11.26	9	16	0.838	11,384
Score of unhealthy eating behavior	Types of unhealthy food consumed in the past 24 h	2.76	0	4	1.518	11,384
Standardized height-for-age z-score	Standardized height-for-age z-score	0.32	−7.55	4.74	1.199	11,384
Score of peer’s dietary and nutritional cognition	peer’s cognition of diet and nutrition	2.22	0	7	1.66	11,384
Score of peer parents’ dietary and nutritional cognition	peer parents’ cognition of diet and nutrition	1.15	0	7	1.834	11,384
Peer father’s education	Educational years of peer’s father	9.4	0	16	2.7	11,384
Peer mother’s education	Educational years of peer’s mother	8.75	0	16	3.196	11,384
Peer parental average education	Average educational years of peer’s parents	9.07	0	16	2.566	11,384
Score of parents’ dietary and nutritional cognition	parents’ cognition of diet and nutrition	1.33	0	7	1.905	11,384
Father’s education	Educational years of father	9.32	0	16	2.982	11,384
Mother’s education	Educational years of mother	8.67	0	16	3.559	11,384
Father’s age	Age of father	38.91	29	55	4.933	11,384
Mother’s age	Age of mother	37.16	29	55	4.791	11,384
Household assets (a index of durable consumer goods used the principal component analysis method [32])	Household durable asset index	0.02	−3.17	0.67	0.43	11,384

## 3. Results

### 3.1. Empirical Model

#### 3.1.1. Theoretical Basis

Various theories such as social influence theory, homogeneity theory and herd effect theory propose the influence of social norms on individual behavior from the perspective of social interaction. Kelman (1958) [33] proposed the social influence theory, which included three forms of social influence: obedience, identity and internalization effects. The obedience effect means that if a person expects to be recognized by others, they will actively respond to people’s requirements or thoughts due to social pressure rather than heartfelt recognition. The identity effect means that if a person wants to establish and maintain close contact with someone or a social group, they not only need to be recognized by the person or the social group but will also be influenced by them. The internalization effect refers to a person sincerely agreeing with the views of others. Latané (1981) [34] advanced the social influence theory and identified three factors affecting people: the number of people who exert influence, the importance of the people who exert influence on the affected and the distance between the people who exert influence and the affected in space and time.

The homogeneity theory refers to similarities between friends, which could be due to social choice and peer influence. The most representative theory in the interpretation of social choice is the similarity-attraction theory, which holds that two people become friends because there are many similarities between them. The socialization theory for peer influence holds that after two people become friends, they affect each other and become similar.

The herd effect theory refers to people’s behavior of blindly conforming to the norm. Asch (1955) [35] found that people’s choices were affected by the group. When an individual’s choices or behaviors are inconsistent with most people, the individual is likely to experience psychological pressure, become anxious and change their previous choices or behaviors.

Peer effect is also a topic of concern in social interaction theory. Peers are the social interaction factor with the highest contact frequency and the longest contact time besides parents, especially for children. It is necessary to discuss the influence of peer effect on students’ nutritional cognition level.

#### 3.1.2. Econometric Model

1. The relationship between peers’ and students’ dietary and nutritional cognition.

To gain an understanding of the correlation between peers’ and students’ dietary and nutritional cognition, the OLS model was defined as:

(1) $$DNCS=\beta_{0}+\beta_{1}\times DNCS_{Peer}+\beta_{2}C+\beta_{3}F+\beta_{4}S+\varepsilon ,$$

where the dependent variable DNCS was the students’ dietary and nutritional cognition score. The score was standardized to estimate the relationship better. $DNCS_{Peer}$ was the peers’ dietary and nutritional cognition score, where the peer was the best friend reported by the students. This was the core variable of the model. *C* represented the students’ characteristics, such as age and gender. F denoted family characteristics, such as parental education and dietary and nutritional cognition. S referred to school effects, included because students’ dietary and nutritional cognition may be correlated with the quality of school facilities and teaching resources. $\beta_{1}$ was the coefficient of interest, as it measured the correlation between peers’ and students’ dietary and nutritional cognition. ε was the random error in normal distribution.

2. The effects of peers’ dietary and nutritional cognition on students’ dietary and nutritional cognition.

Endogeneity problems refer to the correlation between explanatory variables and error terms. The causes of common endogeneity problems include: (1) measurement error, which refers to the error between the value of the explanatory variable used in the model and the real data; (2) omitted explanatory variable, which refers to excluding a relevant variable; (3) simultaneity, which refers to the situation that the explained variable can affect the explanatory variable; (4) selective bias, which refers to the problem of self-selection samples [36]. There might be two endogeneity problems in peer effect research: self-selection and simultaneity bias. Self-selection refers to similar behaviors between two people that may not be caused by one influencing the other but rather because the two people chose to be friends due to similar behavior. Simultaneity bias refers to the inability to judge whether the peer influences the student, or the student influences the peer, which is a mutual causality problem.

- In China, generally, there is only one central primary school in a township or district. For rural and migrant children, the educational administrative system, which is related to the household registration system, guides students to choose schools nearby [37]. Most students will choose the Central Primary School in their township or district to enroll nearby. Otherwise, they need to pay an expensive school-selection fee to go to schools from other townships or districts. However, it is rare for rural and migrant children to pay the fees for choosing a school. Thus, there was no self-selection problem in school choice. In addition, few students indicated diet as the reason for choosing their best friend in the questionnaire. In this study, we designed a question “why choose him/her as your best friend?” We found that when choosing the best friend, 45.38% of the students only considered the friend’s character, 12.71% of the students only considered the friend’s study, 6.04% of the students only considered the friend’s sports ability, 3.02% of the students only considered the friend’s appearance, 11.27% of the students considered the friend’s character, study, sports and appearance at the same time and 21.58% of the students considered other reasons. We then asked, “what other reasons specifically mean”. In the students’ answers, none of the students chose their best friend because of diet. Therefore, there was no self-selection problem in friend choice.
- To address the simultaneity bias, this study used the nutritional cognitive score of peers’ parents as an instrumental variable using the following model:

(2) $$DNCS_{Peer}=\gamma_{0}+\gamma_{1}\times PDNCS_{Peer}+\gamma_{2}\times PEDU_{Peer}+\nu ,$$

where $DNCS_{Peer}$ was the students’ dietary and nutritional cognition score, $PDNCS_{Peer}$ was peers’ parental diet and nutrition cognition score, and $PEDU_{Peer}$ was peers’ parental average education.

3. Effects of Peers’ Dietary and Nutritional Cognition on Dietary Behavior and Physical Attributes.

In order to measure students’ and parents’ dietary nutrition cognition in multiple dimensions, we calculated students’ and parents’ dietary and nutritional cognition scores using item response theory (IRT). This theory can more intuitively reflect the difference in the difficulty of questionnaire questions and distinguish different nutrition cognitive levels [29]. We tested robustness by transforming standardized scores of dietary and nutritional cognition of students and their parents to IRT scores of dietary and nutritional cognition. In the IRT model, each item has an item characteristic curve (ICC), which describes the relationship between the respondent’s ability level and probability of answering the question correctly [38], that is, people with higher nutritional cognition level (or “ability level”) have a higher probability of giving the correct answer, while people with lower ability level have a lower probability of giving the correct answer [39]. 

### 3.2. Empirical Results

#### 3.2.1. Relationship between Peers’ and Students’ Dietary and Nutritional Cognition

Table 2 reports the OLS model results based on Equation (1). The dependent variables of models 1, 2 and 3 were standardized students’ dietary and nutritional cognition scores, with all three models controlling the school fixed effect. The standardized score of peers, dietary and nutritional cognition was the independent variable in model 1. Model 2 included the variables representing students’ personal characteristics, such as gender and age. Model 3 included variables related to family characteristics, such as standardized score of parental dietary and nutritional cognition, father’s education and mother’s education.

Peers’ dietary and nutritional cognition demonstrated a significant positive impact on students’ dietary and nutritional cognition (Table 2). The coefficient of the standardized score of peers’ dietary and nutritional cognition was 0.049, which was statistically significant at the 1% level. This indicated that for each standard deviation increase in the score of peers’ dietary and nutritional cognition, the students’ score of dietary and nutritional cognition increased by 0.049 standard deviations. In addition, there were significant differences in dietary and nutritional cognition between boys and girls. On average, boys’ dietary and nutritional cognition was 0.115 standard deviations lower than that of girls. Furthermore, parental dietary and nutritional cognition and education had a significant impact on students’ dietary and nutritional cognition. The coefficient of the standardized score of parental dietary and nutritional cognition was 0.284, which was significant at the 1% level. The influence of the father’s education on students’ dietary and nutritional cognition was much higher than that of the mother. For every one-year increase in the father’s education, the score of students’ dietary and nutritional cognition increased by 0.016 standard deviations, while for mothers, it increased by 0.009 standard deviations.

#### 3.2.2. Endogenous Analysis

Students’ and peers’ dietary and nutritional cognition may have affected each other, resulting in an endogeneity, such as mutual causality. Therefore, the OLS estimation results may be biased and inconsistent. To address this problem, this study used peers’ parents’ dietary and nutritional cognition and average education as instrumental variables to estimate the result of Equation (2).

A series of tests were conducted on the selected tool variables to determine whether the variables were effective. First, the Cragg-Donald Wald test was used to investigate whether there was a weak identification of instrumental variables. The Cragg–Donald Wald F statistic was 394.553, rejecting the original hypothesis of weak identification (Table 3). Second, the Sargan–Hansen test was used to examine whether there was over-identification of instrumental variables. The *p*-value was 0.116, which accepted the original assumption that all instrumental variables were exogenous variables. Therefore, there was no problem of over-identification. Consequently, instrumental variables were effective.

After solving the endogeneity problem, the peer effect on students’ dietary and nutritional cognition remained significant (Table 3). For each standard deviation of peer dietary nutrition cognition, students’ dietary nutrition cognition improved by 0.086 standard deviations. This result indicated that peers were an important factor affecting students’ dietary nutrition cognition. When the score of peers’ dietary and nutritional cognition increased by one standard deviation, the score of students’ dietary and nutritional cognition increased by 0.086 standard deviations.

#### 3.2.3. Robustness Check

This study tested robustness by changing standardized score of dietary and nutritional cognition of students and their parents to the IRT score of dietary and nutritional cognition of students and their parents. Table 4 shows the results. Column (1) in Table 4 shows the result of OLS regression without considering endogeneity. Column (2) in Table 4 shows the results of IV regression using IRT scores of peer parents’ dietary and nutritional cognition and peer parental average education as instrumental variables. The OLS regression results demonstrated that without considering endogeneity, when the IRT score of peers’ dietary and nutritional cognition increased by one standard deviation, the IRT score of students’ dietary and nutritional cognition increased by 0.043 standard deviations. Moreover, after solving the endogeneity problem, the IV regression results indicated that when peers’ dietary and nutritional cognition IRT score increased by one standard deviation, the students’ dietary and nutritional cognition IRT score increased by 0.079 standard deviations. The results were consistent with the previous conclusions; therefore, the results of this study were robust.

#### 3.2.4. Heterogeneity Analysis

The above analysis assumed that the peer effect on dietary and nutritional cognition was the same for all students. However, the peer effect may vary across individual characteristics, such as friendship, gender, age and school type. In order to test this possibility, this study estimated the peer effect equation using samples separated by friendship (mutual friendship vs. ego-perceived friendship), gender (girls vs. boys), age (10, 11 and 12 years old) and school type (urban migrant children’s school vs. rural public school).

First, there were two kinds of friendships: mutual friendships, where students regarded each other as best friends, and ego-perceived friendships, where only one student regarded the other as their best friend. The peer’s influence on students’ cognition of diet and nutrition varied by friendship. This study conducted a regression on these two sub-samples to explore heterogeneity between them. Columns (1) and (2) in Table 5 show the results of the OLS regression for mutual and ego-perceived friendships without using instrumental variables. Columns (3) and (4) in Table 5 show the results of the IV regression for these two subsamples after solving endogenous problems. The results demonstrated that although the peer effect was no longer significant for mutual friendship students using the IV model, it was close to the significant level according to the standard error. In addition, after solving endogeneity, the coefficient increased. The coefficient of the standardized score of peers’ dietary and nutritional cognition in the IV regression that used the mutual friendship sample was 0.107 (Column 3). The corresponding coefficient in the IV regression that used the ego-perceived sample was only 0.080 (Column 4).

Second, there were significant differences between boys and girls in education. Studies have demonstrated that due to the higher expected return for boys than girls, many families invest more in boys’ education and health [40]. Therefore, this study divided all samples by gender for a heterogeneity analysis. Columns (1) and (2) in Table 6 show the OLS regression results for girls (Column 1) and boys (Column 2). Columns (3) and (4) in Table 6 shows the IV regression results for girls (Column 3) and boys (Column 4). The coefficient of peers’ dietary and nutritional cognition was 0.100, which was significant at the 5% level for girls (column 3). The coefficient of the peers’ dietary and nutritional cognition for boys was 0.083 and insignificant. This suggested that, compared with boys, the peer effect among girls was higher. This finding suggested that girls’ dietary and nutritional cognition was more likely to be influenced by peers.

Third, Piaget’s stage of development theory notes that there are significant differences in cognitive development among children of different ages. Consequently, age is an important variable that affects children’s cognitive abilities. Therefore, this study divided students into three categories by age: 10, 11, and 12 years old. The three samples were regressed to analyze the heterogeneity of age. Columns (1), (2) and (3) in Table 7 show the OLS regression results. Columns (4), (5) and (6) show the IV regression results. The results indicated that the peer effect on students’ dietary and nutritional cognition was heterogeneous at different ages. Specifically, after solving the endogeneity, the influence of peer diet cognition gradually decreased with age. Moreover, Column (6) shows that the peer effect was no longer significant among 12-year-old students. This may be because a 12-year-old child is in the sixth grade of primary school and is at the critical stage of upgrading from primary school to junior high school. Students are constrained by their parents to spend less time with their peers and more time with their parents. Therefore, students may no longer be influenced by their peers.

Finally, with the transfer of rural labor force to cities, the number of migrant children is increasing. In cities, one-fifth of children are migrants from rural areas [41], which has attracted social attention to migrant children. Studies have shown that leaving home can have a negative impact on children. Migrant children lose their original social networks and community service security after leaving their hometowns. Moreover, in the cities where they study, they face difficulties and discrimination in schools, medical treatment, and social security [41]. However, some studies claim that the learning and life experiences of migrant children are conducive to improving their cognitive and non-cognitive abilities [42]. Thus, the impact of migration on children’s physical and mental development is an important and controversial issue. Therefore, this study divided the students into two groups: migrant school students and rural public-school students. Regression was conducted to examine differences in the peer effect on dietary and nutritional cognition among migrant and non-migrant children. Table 8 shows the results of the heterogeneity between urban migrant children’s school students and rural public-school students. Columns (1) and (2) show the OLS regression results, and columns (3) and (4) show the IV regression results. The results demonstrated that, compared with the students in urban migrant children’s schools, the peer effect of rural public-school students on dietary and nutritional cognition was more significant, which indicated that rural public school students were more likely to be affected by their peers’ dietary and nutritional cognition.

## 4. Conclusions

This study offered an alternative explanation for children’s dietary and nutritional cognition by exploring the peer effect as an important factor. In addition, this study addressed the endogeneity problem by using peers’ parents’ dietary and nutritional cognition and average education as instrumental variables. One possible limitation is that there are only seven questions about nutritional cognition in the questionnaire which may not fully represent the nutritional cognition of students or parents. In this study, we find that primary school students’ dietary and nutritional cognition, which was affected by peers’ cognition of diet and nutrition, was an important influencing factor in their dietary behavior and nutritional intake. Furthermore, girls, rural public-school students, younger students and students with ego-perceived friendships were more likely to be influenced by peers’ dietary and nutritional cognition. Finally, we examined the impact of dietary and nutritional cognition of students and their peers on students’ dietary behaviors and physical attributes. The results demonstrated that the dietary and nutritional cognition of students themselves and their peers significantly impacted students’ dietary behavior and physical attributes.

These results provide a basis for school-level interventions and education policies. First, our study shows that the overall nutritional cognition level of primary school students is low at present. Therefore, schools should strengthen the cultivation and education of students’ nutritional cognition. We can select influential students in the class to promote healthy diet and nutrition knowledge and form a good learning environment. It’s also crucial to improve the cognition of rural public-school and lower grade students; second, we should pay attention to the influence of parents’ cognition on students’ nutrition cognition and encourage parents to guide students’ knowledge or behavior of a healthy diet; finally, and more generally, the social returns of any intervention that improves dietary and nutritional cognition may be underestimated if positive spillovers of cognition on other individuals outside the studied environment are neglected.

## Figures and Tables

**Table 2:** Relationship between peer’s dietary and nutritional cognition and student’s dietary and nutritional cognition.

	Model (1)	Model (2)	Model (3)
Standardized score of peer’s dietary and nutritional cognition	0.065 ***	0.059 ***	0.049 ***
	(0.01)	(0.01)	(0.01)
Boy		−0.135 ***	−0.115 ***
		(0.02)	(0.02)
Age		−0.006	0.010
		(0.01)	(0.01)
Standardized score of parents’ dietary nutrition cognition			0.248 ***
			(0.01)
Father’s education			0.016 ***
			(0.00)
Mother’s education			0.009 ***
			(0.00)
Father’s age			0.003
			(0.00)
Mother’s age			0.002
			(0.00)
Household assets			0.028
			(0.02)
Constant	0.274 **	0.413 **	−0.056
	(0.11)	(0.17)	(0.19)
School effects	Yes	Yes	Yes
Observations	11,384	11,384	11,384
R^2^	0.079	0.084	0.116
adjusted R^2^	0.07	0.07	0.11

Note: The value of robust standard errors is reported in parentheses. The definitions for each of the variables are available in Table 1. *** Indicate significance level of 1%. ** Indicate significance level of 5%.

**Table 3:** Effects of peer’s dietary and nutritional cognition on student’s dietary and nutritional cognition.

	Standardized Score of Student’s Dietary and Nutritional Cognition
Standardized score of peer’s dietary and nutritional cognition	0.084 **
	(0.04)
Boy	−0.110 ***
	(0.02)
Age	0.010
	(0.01)
Standardized score of parents’ dietary nutrition cognition	0.246 ***
	(0.01)
Father’s education	0.015 ***
	(0.00)
Mother’s education	0.009 ***
	(0.00)
Father’s age	0.003
	(0.00)
Mother’s age	0.002
	(0.00)
Household assets	0.027
	(0.02)
Constant	−0.036
	(0.18)
School effects	Yes
Observations	11,384
R^2^	0.115
adjusted R^2^	0.11
Cragg-Donald Wald F statistic	394.438
Sargan test statistic	2.024
Sargan test *p* value	0.155

Note: The value of robust standard errors is reported in parentheses. The definitions for each of the variables are available in Table 1. *** Indicates significance level of 1%. ** Indicates significance level of 5%.

**Table 4:** Robustness check.

	OLS	IV
IRT score of peer’s dietary and nutritional cognition	0.043 ***	0.079 **
	(0.01)	(0.03)
Boy	−0.088 ***	−0.084 ***
	(0.01)	(0.01)
Age	0.006	0.007
	(0.01)	(0.01)
IRT score of parents’ dietary nutrition cognition	0.219 ***	0.216 ***
	(0.01)	(0.01)
Father’s education	0.010 ***	0.010 ***
	(0.00)	(0.00)
Mother’s education	0.006 ***	0.006 ***
	(0.00)	(0.00)
Father’s age	0.002	0.001
	(0.00)	(0.00)
Mother’s age	0.002	0.002
	(0.00)	(0.00)
Household assets	0.019	0.019
	(0.01)	(0.01)
Constant	0.004	0.019
	(0.13)	(0.12)
School effects	Yes	Yes
Observations	11,384	11,384
R^2^	0.113	0.112
adjusted R^2^	0.10	0.10
Cragg-Donald Wald F statistic		423.793
Sargan test statistic		1.736
Sargan test *p* value		0.188

Note: The value of robust standard errors is reported in parentheses. The definitions for each of the variables are available in Table 1. *** Indicate significance level of 1%. ** Indicate significance level of 5%.

**Table 5:** Peer effect on the cognition of diet and nutrition for students with mutual friendship or ego-perceived friendship.

	OLS		IV	
	Mutual Friendship	Ego-Perceived Friendship	Mutual Friendship	Ego-Perceived Friendship
Standardized score of peer’s dietary and nutritional cognition	0.068 ***	0.029 **	0.107	0.080 *
	(0.02)	(0.01)	(0.08)	(0.05)
Boy	−0.105 ***	−0.104 ***	−0.101 ***	−0.099 ***
	(0.03)	(0.02)	(0.03)	(0.02)
Age	0.001	0.011	0.000	0.011
	(0.02)	(0.01)	(0.02)	(0.01)
Standardized score of parents’ dietary nutrition cognition	0.261 ***	0.236 ***	0.259 ***	0.233 ***
	(0.02)	(0.02)	(0.02)	(0.02)
Father’s education	0.018 ***	0.014 ***	0.017 ***	0.014 ***
	(0.01)	(0.00)	(0.01)	(0.00)
Mother’s education	0.006	0.011 ***	0.006	0.010 ***
	(0.00)	(0.00)	(0.00)	(0.00)
Father’s age	0.004	0.002	0.004	0.002
	(0.00)	(0.00)	(0.00)	(0.00)
Mother’s age	0.000	0.004	0.000	0.004
	(0.00)	(0.00)	(0.00)	(0.00)
Household assets	0.064 **	−0.004	0.064 **	−0.005
	(0.03)	(0.03)	(0.03)	(0.03)
Constant	−0.132	−0.051	−0.132	−0.011
	(0.31)	(0.24)	(0.29)	(0.23)
School effects	Yes	Yes	Yes	Yes
Observations	4783	6601	4783	6601
R^2^	0.135	0.119	0.133	0.117
adjusted R^2^	0.11	0.10	0.11	0.10
Cragg-Donald Wald F statistic			90.947	282.140
Sargan test statistic			1.705	0.541
Sargan test *p* value			0.192	0.462

Note: The value of robust standard errors is reported in parentheses. The definitions for each of the variables are available in Table 1. *** Indicate significance level of 1%. ** Indicate significance level of 5%. * Indicate significance level of 10%.

**Table 6:** Peer effect on the cognition of diet and nutrition for girls and boys.

	OLS		IV	
	Girls	Boys	Girls	Boys
Standardized score of peer’s dietary and nutritional cognition	0.077 ***	0.011	0.097 *	0.081
	(0.01)	(0.01)	(0.05)	(0.05)
Age	0.012	0.008	0.012	0.009
	(0.02)	(0.02)	(0.02)	(0.02)
Standardized score of parents’ dietary nutrition cognition	0.257 ***	0.247 ***	0.255 ***	0.244 ***
	(0.02)	(0.02)	(0.02)	(0.02)
Father’s education	0.020 ***	0.012 **	0.020 ***	0.012 **
	(0.01)	(0.00)	(0.01)	(0.00)
Mother’s education	0.008 *	0.010 **	0.008 *	0.010 **
	(0.00)	(0.00)	(0.00)	(0.00)
Father’s age	0.003	0.002	0.003	0.001
	(0.00)	(0.00)	(0.00)	(0.00)
Mother’s age	0.003	0.003	0.003	0.003
	(0.00)	(0.00)	(0.00)	(0.00)
Household assets	0.033	0.028	0.032	0.029
	(0.03)	(0.03)	(0.03)	(0.03)
Constant	0.047	−0.226	0.063	−0.184
	(0.26)	(0.26)	(0.26)	(0.25)
School effects	Yes	Yes	Yes	Yes
Observations	5382	6002	5382	6002
R^2^	0.135	0.114	0.135	0.110
adjusted R^2^	0.11	0.10	0.11	0.09
Cragg-Donald Wald F statistic			193.080	196.966
Sargan test statistic			1.423	0.453
Sargan test *p* value			0.233	0.501

Note: The value of robust standard errors is reported in parentheses. The definitions for each of the variables are available in Table 1. *** Indicates significance level of 1%. ** Indicates significance level of 5%. * Indicates significance level of 10%.

**Table 7:** Peer effect on the cognition of diet and nutrition for students with different ages.

	OLS			IV		
	10 Years Old	11 Years Old	12 Years Old	10 Years Old	11 Years Old	12 Years Old
Standardized score of peer’s dietary and nutritional cognition	0.064 **	0.041 ***	0.048 ***	0.178 **	0.160 ***	0.025
	(0.03)	(0.02)	(0.02)	(0.09)	(0.06)	(0.07)
Boy	−0.130 ***	−0.099 ***	−0.130 ***	−0.118 ***	−0.080 ***	−0.133 ***
	(0.04)	(0.03)	(0.03)	(0.04)	(0.03)	(0.03)
Standardized score of parents’ dietary nutrition cognition	0.215 ***	0.248 ***	0.282 ***	0.210 ***	0.241 ***	0.283 ***
	(0.03)	(0.02)	(0.03)	(0.03)	(0.02)	(0.03)
Father’s education	0.009	0.008	0.027 ***	0.008	0.007	0.027 ***
	(0.01)	(0.01)	(0.01)	(0.01)	(0.01)	(0.01)
Mother’s education	0.007	0.012 ***	0.008	0.007	0.012 ***	0.008
	(0.01)	(0.00)	(0.01)	(0.01)	(0.00)	(0.01)
Father’s age	0.003	0.002	0.000	0.004	0.002	0.001
	(0.01)	(0.00)	(0.01)	(0.01)	(0.00)	(0.01)
Mother’s age	0.003	−0.001	0.008 *	0.002	−0.000	0.008
	(0.01)	(0.00)	(0.01)	(0.01)	(0.00)	(0.01)
Household assets	0.037	0.035	0.067	0.033	0.033	0.066
	(0.05)	(0.03)	(0.04)	(0.05)	(0.03)	(0.04)
Constant	−0.090	0.212	0.048	−0.052	0.270	0.015
	(0.34)	(0.20)	(0.25)	(0.29)	(0.19)	(0.26)
School effects	Yes	Yes	Yes	Yes	Yes	Yes
Observations	1982	4971	3681	1982	4971	3681
R^2^	0.188	0.127	0.132	0.178	0.115	0.132
adjusted R^2^	0.13	0.10	0.10	0.12	0.09	0.10
Cragg-Donald Wald F statistic				78.986	168.819	105.391
Sargan test statistic				2.611	1.453	0.017
Sargan test *p* value				0.106	0.228	0.895

Note: The value of robust standard errors is reported in parentheses. The definitions for each of the variables are available in Table 1. *** Indicates significance level of 1%. ** Indicates significance level of 5%. * Indicates significance level of 10%.

**Table 8:** Peer effect on the cognition of diet and nutrition for students in urban migrant children’s school or rural public-school.

	OLS		IV	
	Urban Migrant Children’s School	Rural Public-School	Urban Migrant Children’s School	Rural Public-School
Standardized score of peer’s dietary and nutritional cognition	0.025	0.058 ***	0.343	0.075 *
	(0.02)	(0.01)	(0.23)	(0.04)
Boy	−0.100 ***	−0.120 ***	−0.077 *	−0.117 ***
	(0.04)	(0.02)	(0.04)	(0.02)
Age	0.019	0.007	0.039	0.007
	(0.02)	(0.01)	(0.03)	(0.01)
Standardized score of parents’ dietary nutrition cognition	0.000	0.247 ***	0.000	0.246 ***
	(0.00)	(0.01)	(0.00)	(0.01)
Father’s education	0.007	0.018 ***	0.007	0.018 ***
	(0.01)	(0.00)	(0.01)	(0.00)
Mother’s education	0.015 **	0.007 **	0.012	0.007 **
	(0.01)	(0.00)	(0.01)	(0.00)
Father’s age	0.009	0.001	0.005	0.001
	(0.01)	(0.00)	(0.01)	(0.00)
Mother’s age	−0.005	0.005	−0.003	0.005
	(0.01)	(0.00)	(0.01)	(0.00)
Household assets	−0.011	0.067 **	−0.021	0.067 **
	(0.03)	(0.03)	(0.03)	(0.03)
Constant	−0.276	−0.459 **	−0.216	−0.465 **
	(0.34)	(0.20)	(0.35)	(0.21)
School effects	Yes	Yes	Yes	Yes
Observations	2788	8596	2788	8596
R^2^	0.053	0.135	−0.041	0.135
adjusted R^2^	0.04	0.13	−0.05	0.12
Cragg-Donald Wald Fstatistic			21.342	405.893
Sargan test statistic			0	0.884
Sargan test *p* value				0.347

Note: The value of robust standard errors is reported in parentheses. The definitions for each of the variables are available in Table 1. *** Indicates significance level of 1%. ** Indicates significance level of 5%. * Indicates significance level of 10%.

## Data Availability

The data presented in this study are available on request from the corresponding author. The data are not publicly available due to ethical.

## Appendix A

**Table A1:** The questions related to diet and nutrition cognition.

Question	Options
1. Which of the following states do you consider to be healthy? (Single topic selection)	1. someone’s health was nothing wrong 2. someone has good physical performance 3. someone has strong physical strength 4. A person is not only free of disease, but also has good psychological and social adaptability 5. other
2. What is the best food source of vitamins and minerals? (Single topic selection)	1. legumes, dairy 2. grains 3. fresh vegetables and fruits 4. meat, eggs 5. unknown
3. What is the foods with the highest protein content? (Single topic selection)	1. Dairy 2. grains 3. vegetables and fruits 4. meat and eggs 5. do not know
4. Which of the following foods are the best sources of calcium? (Single topic selection)	1. beans, milk 2. grains 3. vegetables and fruits 4. meat, eggs 5. do not know
5. Which do you think will help growing taller? (Can choose more)	1. Drink more milk 2. increase exercise time 3. proper exposure to the sun 4. eat more carrots 5. do not know
6. How to prevent iron deficiency anemia through diet? (Single topic selection)	1. eat meat and fresh vegetables and fruits 2. drink more milk 3. eat bland food 4. do not know
7. What disease does salty food often cause easily? (Single topic selection)	1. Diabetes 2. hypertension 3. gastritis 4. do not know
